# Risk Factors for Mortality in Patients With Cerebral Palsy: A Systematic Review and Meta-Analysis

**DOI:** 10.7759/cureus.39327

**Published:** 2023-05-22

**Authors:** Sarah S Aldharman, Fahad S Alhamad, Rahaf M Alharbi, Yousef S Almutairi, Mhd Walid M Alhomsi, Saeed A Alzahrani, Abdulaziz S Alayyaf, Norah H Alabdullatif, Suaad S Bin Saeedu, Saud A Alnaaim

**Affiliations:** 1 College of Medicine, King Saud bin Abdulaziz University for Health Sciences, Riyadh, SAU; 2 College of Medicine, Almaarefa University, Riyadh, SAU; 3 College of Medicine, Prince Sattam bin Abdulaziz University, Al-Kharj, SAU; 4 College of Medicine, Sulaiman Alrajhi University, Al-Qassim, SAU; 5 Collage of Medicine, Prince Sattam bin Abdulaziz University, Al-Kharj, SAU; 6 College of Medicine, Qassim University, Al-Qassim, SAU; 7 Collage of Medicine, Alfaisal University, Riyadh, SAU; 8 Department of Clinical Neurosciences, King Faisal University, Al-Ahsa, SAU

**Keywords:** meta-analysis, systematic review, cerebral palsy, mortality, risk factors

## Abstract

Cerebral palsy (CP) is a developmental and physical disorder with different degrees of severity. Since CP manifests itself in early childhood, numerous research studies have concentrated on children with CP. Patients with CP encounter different severity of motor impairments attributed to the damage or disturbance to the fetal or infant developing brain, which begins in early childhood and persists through adulthood. Patients with CP are more prone to mortality compared to the general population. This systematic review and meta-analysis aimed to assess the risk factors that predict and influence mortality in patients with CP. Systematic search for studies assessing the risk factors for mortality in CP patients that were conducted from 2000 to 2023 in Google Scholar, PubMed, and Cochrane Library was performed. R-One Group Proportion was used for statistical analysis and Newcastle-Ottawa Quality Assessment Scale (NOS) for quality appraisal. Of the 1791 total database searches, nine studies were included. Based on the NOS tool for quality appraisal, seven studies were of moderate quality, and two studies were rated as of high quality. The risk factors included pneumonia and other respiratory infections, neurological disorders, circulatory diseases, gastrointestinal infections, and accidents. Pneumonia (OR = 0.40, 95% CI = 0.31 - 0.51), neurological disorders (OR = 0.11, 95% CI = 0.08 - 0.16), respiratory infections (OR = 0.36, 95% CI = 0.31 - 0.51), cardiovascular and circulatory diseases (OR = 0.11, 95% CI = 0.04 - 0.27), gastrointestinal and metabolic causes (OR = 0.12, 95% CI = 0.06 - 0.22), and accidents (OR = 0.05, 95% CI = 0.04 - 0.07) were the risk factors assessed. It was concluded that multiple factors predict the risk of mortality in patients with CP. Pneumonia and other respiratory infections are associated with a high risk of mortality. Cardiovascular and circulatory diseases, gastrointestinal and metabolic disorders, and accidents are strongly linked to mortality in CP patients.

## Introduction and background

Cerebral palsy (CP) is a developmental and physical disorder with different degrees of severity but with common developmental characteristics commencing early on in life and persisting throughout an individual’s life [1-3]. CP is a neurological syndrome that defines a group of permanent malfunctions in the development of movement and posture, leading to activity limitation [2,3]. The condition is attributed to non-progressive disturbances or damage to the developing fetal or infant brain and is the most prevalent form of disability in children [1]. CP is commonly classified depending on the type of associated movement disorder. Based on this mode of classification, an individual may have one or a combination of the following conditions: spastic (stiff muscles), dyskinesia (uncontrollable movements), ataxia (poor balance and coordination), and hypotonic (muscle tone decrease) [4,5]. Often, children with CP experience several health complications and functions, including underdeveloped and weak bones and muscles, limited physical activity levels, excess abdominal fat, musculoskeletal depots, mental health disorders, and communication impairments [1]. Experiencing health and function disorders in childhood increases the chance of developing chronic diseases as well as other mental health disorders (such as depression and anxiety), which may cause premature mortality. 

Since CP manifests itself in early childhood, much research has concentrated on children with CP. Population-based registries of CP, majorly in Europe and Australia, have historically revealed CP prevalence ranging between 1.5 and 2.5 per 1,000 live births. Recent studies in the United States [6], Taiwan [7], and Egypt [8] have, however, reported a prevalence rate of more than 3 in 1,000 live births in individuals 4 to 48 years. Recently, researchers have shifted their focus to the impact of CP and its associated secondary consequences later in life. As CP is a motor disorder, it has been observed that individuals with CP, right from childhood, show elevated sedentary lifestyles and limited physical activity that only soars throughout the transition to adolescence and adulthood, especially in those who are nonambulatory [9,10]. It is imaginable that the occurrence of such a disorder affecting neurological behavior or control of both voluntary and involuntary movements may have health implications. The diagnosis of CP is based on a combination of the neurologic examination, neuroimaging imaging results, and identification of clinical risk factors. Therefore, diagnosis is always complicated and delayed and often happens between the age of 1 to 2 years or after [11]. Recently, diagnosing CP at an earlier age and more accurately has been possible. This is desirable since it enables early initiation of therapeutic treatment that may optimize long-term outcomes during rapid brain development and neuroplasticity [12]. The pathway to CP diagnosis varies depending on whether a child has observable risk factors for CP during the neonatal period, which prompts early screening and special or closer developmental monitoring. Novak and colleagues have suggested that infants with newborn-identifiable risk factors for CP must undergo a standardized neurological assessment, motor examination, and neuroimaging to help make an early diagnosis, desirably not later than five months after birth [12].

On the other hand, the diagnosis of CP in children with no identifiable risk factors often occurs later in life after parents or pediatricians discover that they are not successfully achieving their required motor development as expected. Signs that necessitate specific examination for CP include the inability to sit by nine months, limb preference or presence of symmetric movements, or the lack of ability to support the weight on the plantar surface of the feet [12]. This assessment should comprise a standard neurological evaluation, a neuromotor examination, and, when possible, magnetic resonance imaging neuroimaging to observe brain lesions that may support the diagnosis [2].

A better understanding of the risk factors of mortality in patients with CP could encourage improvement in the medication and prescription strategies that improve treatment and management of health complications to prolong life. Therefore, this study aimed to explore the risk factors for mortality in patients with CP.

## Review

Materials and methods

Study Design and Literature Search

This study is a systematic review and meta-analysis of nine primary studies that adhered to the Preferred Reporting Items for Systematic Reviews and Meta-Analyses (PRISMA) model of reporting guidelines [13]. An exhaustive systematic literature search was carried out in electronic databases to examine the risk factors for mortality in patients with CP. The searched databases include Google Scholar, PubMed, and Cochrane Library for articles published in English from 2000 to 2023 exploring the risk factors for mortality in patients with CP.

Search Strategy

Key terms were used in combination with the Boolean expressions “AND” and “OR.” The following keywords or phrases were used during the search: “risk factors of mortality” OR “causes of mortality” AND “patients with CP” OR “CP.” The search was customized to include only full-text articles published in English between 2000 and 2023. References list from the studies included were searched manually to find more studies that might not have been captured by the initial search strategy.

Eligibility Criteria

The following inclusion criteria were applied to select related studies for this analysis: first, primary studies assessing the risk factors for death or the underlying causes of death in patients with CP at any age in any part of the world. Second, studies that assessed the rate or comparative risk factors for mortality in patients with CP. Third, empirical studies published in English between 2000 and 2023.

The following exclusion criteria were used to exclude studies: first, secondary sources including other systematic reviews, case reports, abstracts and magazines, and newspapers reporting on the risk factors of death for patients with CP. Second, studies published in a non-English language before 2000.

Data Extraction and Quality Appraisal

The selection in and selection and extraction of evidence from the studies that merited the inclusion criteria were done by two independent reviewers. Any discrepancies, if necessary, were resolved through consultation with a third author. The quality of the studies included studies was assessed based on the Newcastle-Ottawa Quality Assessment Scale (NOS) [14]. High-quality sources were chosen according to the selection criteria and marked with a star.

Statistical Analysis

Data analysis was done using R-One Group Proportion meta-analysis according to the requirements of the data retrieved. The heterogeneity of the studies included was accounted for using the I2. If the I2 statistic had a value < 50%, then there was no significant heterogeneity present. A significant heterogeneity value was shown by I2 ≥50%. The random-effect model was used to determine the pooled 95%CI. 

Results

Search Results

**Figure 1:**
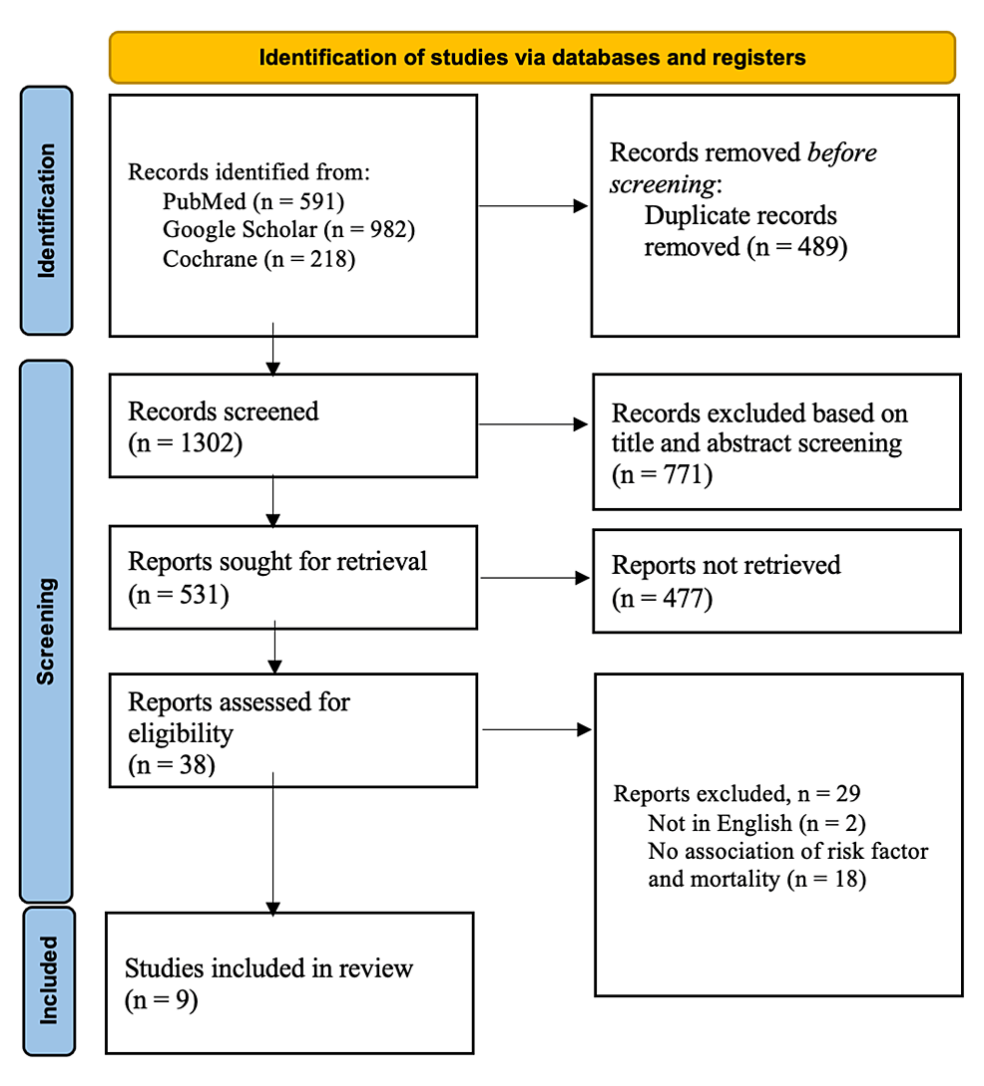
PRISMA flow chart for the study selection process PRISMA: Preferred Reporting Items for Systematic Reviews and Meta-Analyses

The database searches discovered 1791 studies, out of which 489, being duplicates, were removed. After screening for titles and abstracts, 771 studies were excluded. The remaining 531 studies were sought for retrieval, but 477 could not be successfully retrieved. The full text of 38 studies was examined for eligibility; 29 were excluded since they failed to satisfy the inclusion criteria outlined earlier, while the other nine studies were included. A manual search was done for the reference list of the studies included, but no additional studies were found. Although seven conference abstract publications were reviewed, they were not added to the final selection due to insufficient data (i.e., they did not present values for risk factors and mortality trends in patients with CP). Therefore, a total of nine studies were included in this study. The study selection process is illustrated in Figure 1. The characteristics of the studies included are shown in Table 1.

**Table 1:** Characteristics of the studies included P: Pneumonia, RC: respiratory causes (failure, aspiration, infection, and arrest), Ep: epilepsy, Gst: gastrointestinal problems, CP: cerebral palsy, Cd: circulatory disease, Cdc: cardiovascular causes, ESP: elective surgical procedures, Ac: accidents, OR: Odds ratio, CI: confidence interval, MR: mortality rate, CoDs: causes of death

Study	Location	Objective	Study design	Follow-up period	Number of Patients with CP	Mortality	Risk factors for mortality/Causes of Death
Baird et al., 2011 [15].	South East Thames Region	To determine mortality and predictors from age 1 to 18.	Retrospective Cohort study	1989 - 1992	n = 346 children with bilateral cerebral palsy (CP).	61(17.94%)	P (19) RC (20) Ep (8) Pancreatitis (1) Diabetic ketoacidosis (1) hepatic failure (1) renal failure (1) Gst (4) CP (6)
Blair et al., 2019 [16].	Western Australia	To study survival patterns and mortality of people with CP based on their impairments.	Retrospective study		n = 3185	436 (13.7%)	RC 56.8% (187) No sufficient immediate cause listed 7.3% (24) Ep 6.7% (29) Ac/trauma 6.4% (21) Non-RC 4.9 (16) Cancer 4.0% (13) Cd 3.6% (12) Major organ failure 3.0% (10 > 1 sufficient cause 3.0% (10) Miscellaneous 2.7% (9) Congenital disabilities 1.5% (5) Unknown cause (10)
Himmelmann and Sundh, 2015 [17].	Western Sweden	To research CoDs and survival with CP type and associated motor impairment.	Clinical Observational	2002 to 2009	n = 1856 1033 males 823 females	180 (9.6%)	Neurological causes (21) Cdc (26) RC and P (96) Gst and metabolic causes (17) Ac (11) Non-P infections (4) malignant tumors (leukemia) (5)
Jahan et al., 2019 [18].	Bangladesh	To determine the mortality rate, CoD, and death predictors in children with CP Bangladesh.	Prospective	January 2015 – December 2016	678 children	29	Meningitis 9 (31%) P 11 (28%) Sepsis 4 (14%) Gst. 2 (7%) Unspecified infectious disease 1 (3%) Cdc 1 (3%) Ac 2 (7%) Unknown 1 (3%)
Namaganda et al., 2020 [19].	Uganda	To determine risk variables, CODs, and the MR of children with CP in Uganda.	Longitudinal	2015 – 2019	97 children (2–17 years)	15	Anemia/malnutrition 6 (40%) Other infections 5 (33%) Malaria 4 (27%)
Prastiya et al., 2018 [20].	Surabaya, Indonesia	To look at the mortality risk factors for CP in Indonesian children.	Observational analytic	January 2014 – December 2016	55 children	12	P: OR = 5.185, 95%CI = 1.249- 21.52 AKI: OR = 3.333, 95%CI = 1.317- 8.436 GMFCS: OR = 1.480, 95%CI = 1.184- 1.850 Acute Diarrhea: OR = 0.262, 95%CI =0.015-4.529 Meningoencephalitis: OR = 0.583, 95%CI =0.493- 4.563 Ep: OR = 3.143, 95%CI = 0.747- 13.22 Subdural hygroma: OR = 1.308, 95%CI =1.123- 1.523 Leukemia: OR = 4.909, 95%CI = 2.897- 8.318 Sepsis OR = 0.225, 95%CI = 0.039- 1.303
Reddihough et al., 2001 [21].	Victoria, Australia	To research the features of CP-affected children and their causes of mortality	Retrospective	1970 – 1995		155	P (69), Sepsis (7) Unexpectedly deaths (7) Ep (6) ESP (2) Miscellaneous (7) Unknown (65)
Reid et al., 2012 [22].	Victoria, Australia	To investigate the mortality rates, factors, trends, and causes for CP patients.	Prospective	1970 to 2004	n = 3507 1972 males 1535 females	418	No CoDs listed (42) Missing data (40) CP (207) Perinatal factors (11) Birth defects (42) Scoliosis (15) Ep (68) RC in 107 of 287 severe motor impairment
Ryan et al., 2019 [23].	England	To assess the Cdc, cancer, and RC mortality rates among persons with CP.	Cohort study	1998 – 2015	n=958 Males 403 Females 455	142	Observed/Expected Malignant neoplasms 13/9.1 Cd 28/8.8 Cdc 9/5.0 Cerebrovascular diseases 8/2.3 RC 38/2.8

Quality Assessment

Based on the NOS tool for quality appraisal as shown in Table 2, seven studies were of moderate quality, two studies were rated as of high quality, and none of the studies was of low quality.

**Table 2:** Quality appraisal using the NOS NOS: Newcastle-Ottawa Quality Assessment Scale

Study	Selection (Max 4)	Comparability (Max 2)	Outcome (Max 3)	Total Score	Quality
Baird et al., 2011 [15].	3	1	2	6	Moderate
Blair et al., 2019 [16].	3	1	2	6	Moderate
Himmelmann and Sundh, 2015 [17].	3	1	3	7	High
Jahan et al., 2019 [18].	3	1	2	6	Moderate
Namaganda et al., 2020 [19].	2	1	2	5	Moderate
Prastiya et al., 2018 [20].	3	1	2	6	Moderate
Reddihough et al., 2001 [21].	2	1	2	5	Moderate
Reid et al., 2012 [22].	2	1	2	5	Moderate
Ryan et al., 2019 [23].	3	1	3	7	High

Risk Factors for Mortality in Patients With CP

**Figure 2:**
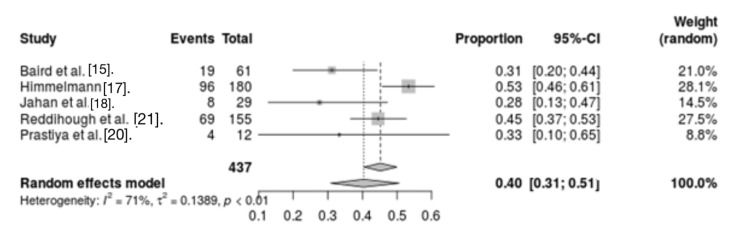
Forest plot for pneumonia as a CP mortality risk factor CP: Cerebral palsy

Pneumonia: Five studies evaluating the association between pneumonia and mortality in CP patients were screened. The results indicated significant heterogeneity between studies (I2 = 71%, p < 0.01). Analysis of these studies showed that pneumonia was a risk factor for mortality in CP patients (OR = 0.40, 95% CI = 0.31 - 0.51) (Figure 2).

**Figure 3:**
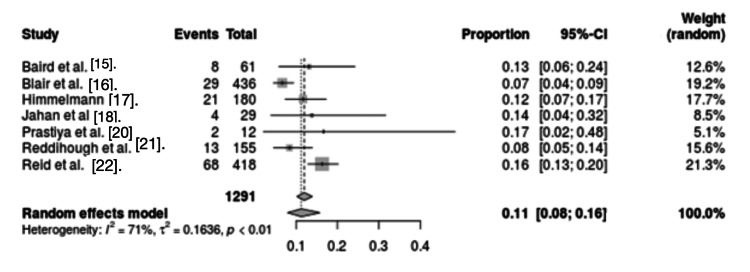
Forest plot for neurological disorders and sepsis as a mortality risk factor

Neurological disorders and sepsis: Seven studies assessed the association of neurological disorders and sepsis with mortality in CP patients. The results showed significant heterogeneity between studies (I2 = 71%, p < 0.01). Analysis of these studies confirmed that neurological disorders and sepsis were risk factors for mortality in CP patients (OR = 0.11, 95% CI = 0.08 - 0.16) (Figure 3).

**Figure 4:**
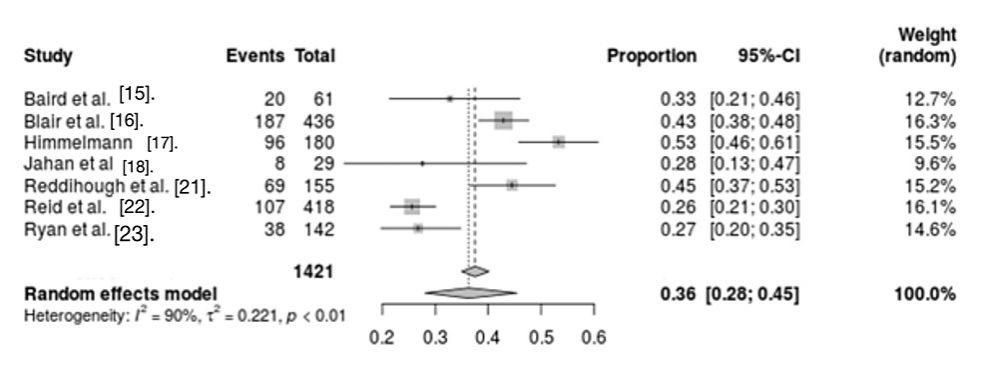
Forest plot for respiratory causes as mortality risk factors for CP CP: Cerebral palsy

Respiratory infections: Seven studies evaluated the association between respiratory infections and mortality in CP patients. The analysis revealed significant heterogeneity between studies (I2 = 90%, p < 0.01). This analysis determined respiratory diseases as a risk factor for mortality in CP patients (OR = 0.36, 95% CI = 0.31 - 0.51) (Figure 4).

**Figure 5:**
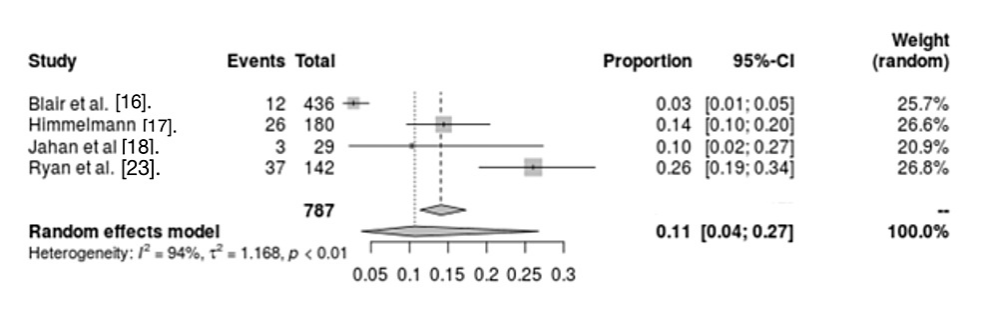
Forest plot for cardiovascular and circulatory diseases as a risk factor

Cardiovascular and circulatory diseases: Four studies evaluated the relationship between cardiovascular and circulatory diseases and mortality in CP patients. The analysis showed significant heterogeneity between studies (I2 = 94%, p < 0.01). The analysis of these four studies revealed cardiovascular and circulatory disorders were a risk factor for mortality in CP patients (OR = 0.11, 95% CI = 0.04 - 0.27) (Figure 5).

**Figure 6:**
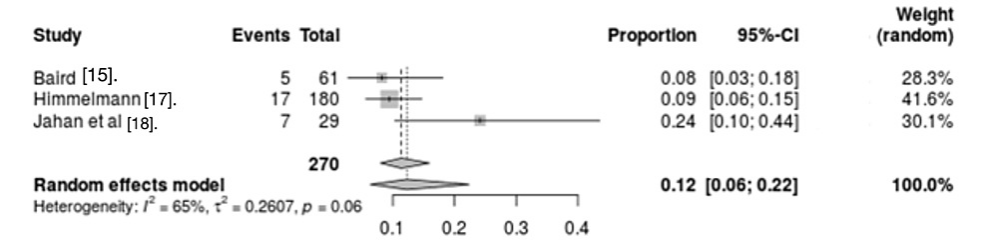
Forest plot for gastrointestinal and metabolic disorders as a risk factor

Gastrointestinal and metabolic causes: Three studies screened for the association between gastrointestinal and metabolic causes and mortality in CP patients. The results indicated significant heterogeneity between studies (I2 = 65%, p = 0.06). Analysis results of the three studies revealed that gastrointestinal and metabolic disorders were mortality risk factors for CP patients (OR = 0.12, 95% CI = 0.06 - 0.22) (Figure 6).

**Figure 7:**
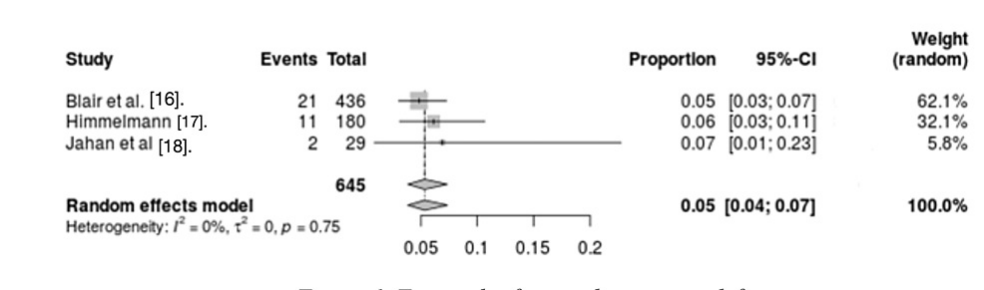
Forest plot for accidents as a risk factor

Accidents: Three studies assessed the association between accidents and mortality in CP patients. The results showed no significant heterogeneity between studies (I2 = 0%, p = 0.75). Analysis of these studies showed that accidents were a risk factor for mortality in CP patients (OR = 0.05, 95% CI = 0.04 - 0.07) (Figure 7).

Table 3 summarizes the meta-analysis results for causes of death in patients with CP.

**Table 3:** Summary of meta-analysis results for causes of death in patients with CP CP: Cerebral palsy

Risk factor/cause of death	Random-effect model [OR (95% CI)]
Pneumonia	0.40 (0.31 – 0.51)
Neurological disorders and sepsis	0.11 (0.08 – 0.16)
Respiratory causes	0.36 (0.31 – 0.51)
Cardiovascular and circulatory diseases	0.11 (0.04 – 0.27)
Gastrointestinal and metabolic causes	0.12 (0.06 – 0.22)
Accidents	0.05 (0.04 – 0.07)

Discussion

This systematic review and meta-analysis aimed at identifying the risk factors for or causes of death in patients with CP. In this review, an analysis of nine cohort studies of 10837 patients with CP, 1448 deaths were recorded, accounting for 13.36% of the CP patients. The causes of death pooled across the studies used in this review include pneumonia and other respiratory causes, neurological disorders, sepsis, cardiovascular and circulatory diseases, gastrointestinal and metabolic causes, and accidents (for example, fires and accidental drowning). The outcomes of this study confirm that patients with CP are at a higher risk of death due to pneumonia (p < 0.01) or other respiratory causes (including respiratory failure, lower respiratory tract infections or influenza, asphyxiation/obstruction, pneumonitis, acute bronchiolitis, and asthma) (p < 0.01), neurological disorders and sepsis (p < 0.01), and cardiovascular and circulatory diseases (p < 0.01). Ischemic heart disease (angina, myocardial, and ischemia infarction), stroke (cerebrovascular disease), heart, and peripheral vascular disease are the most common but preventable cardiovascular diseases [24]. This study also attributed gastrointestinal causes and accidents as mortality risks or immediate causes of death in patients with CP. However, statistical analysis showed that these risk factors were insignificant causes of mortality in patients with CP (i.e., p > 0.05).

Seven studies included in this review and analysis attributed a significant proportion of the reported deaths in CP patients to respiratory infections [15-18, 21-23]. Still, they did not describe the exact nature of such problems. Previous studies have also cited respiratory causes of mortality as the most common among individuals with CP [21,22,25]. Most of these studies recognize aspiration as linked with oropharyngeal dysfunction, resulting in pneumonia as a critical factor. This is consistent with the findings of a recent study estimating the hospital admissions of CP patients due to respiratory problems, which also included gastro-esophageal reflux problems, seizures, and past respiratory diseases, mainly those requiring antibiotics or hospitalization, but excluding scoliosis [17]. These factors demonstrate that those who suffer from chronic lung disease are predisposed to recurrent respiratory failure or Pneumonia observed in CP individuals with oropharyngeal dysfunction. Eventually, it manifests as an extreme degree of susceptibility or risk to respiratory disease accompanied by repeated and prolonged hospital admissions for respiratory support, which may require non-inversive ventilation or oxygen [26]. For some individuals, this may be experienced in childhood. At the same time, other studies have suggested that it may be delayed increasingly until young adulthood and may come with the transition from child care into adult care with compromised care due to limited ready access to familiar care physicians [27]. Many of the immediate causes of death identified are preventable or treatable through appropriate health care services, timely intervention measures, and infection management, which require appropriately designed intervention programs, training, and raising of awareness.

Despite the concerted effort, this study has some limitations at the individual study level as well as at the meta-analysis level. At a meta-analysis level, considerable heterogeneity was noticed in the collective estimate of the mortality risk. Also, to quantify risk factors associated with mortality in patients with CP, unadjusted and adjusted data available from individual studies were used for pooling. Unfortunately, there was inconsistent reporting in multivariable analysis with adjustments for different confounding risk factors or variables across studies, thus limiting the conclusions that can be arrived at from these observations. It is acknowledged that the pooling of unadjusted estimates cannot account for confounding variables. In addition, the risk factors identified may not necessarily, in isolation, be the underlying cause of increased mortality but are instead linked to other increased mortality due to their link to other conditions or disease features of CP. Moreover, there were insufficient evidence data to gather risks of mortality for patients with CP because of inconsistent reporting. At the individual study level, there was limited information on how the confounding variables increase mortality in patients with CP and how they modify the course of the disorder.

Following this review, it is recommended to implement proper preventive and management measures against the risk factors associated with CP mortality. We suggest future research to focus on prospective cohort studies investigating these risk factors and the patient's comorbidities that could be linked to the high mortality rates.

## Conclusions

CP has a high risk of death and morbidity. This review and meta-analysis illustrated the effects of various characteristics of the risks for mortality in patients with CP. The significant risk factors and causes of death in patients with CP include pneumonia and other respiratory diseases, neurological diseases, and cardiovascular diseases. Other causes include gastrointestinal and metabolic disorders and accidents (drowning or fires). These findings may help predict and improve the prognosis of patients with CP.
